# Very small embryonic-like stem cells are the elusive mouse endometrial stem cells- a pilot study

**DOI:** 10.1186/s13048-015-0138-2

**Published:** 2015-03-11

**Authors:** Pranesh Gunjal, Deepa Bhartiya, Siddhanath Metkari, Dhananjay Manjramkar, Hiren Patel

**Affiliations:** Stem Cell Biology Department, National Institute for Research in Reproductive Health, Mumbai, 400 012 India; Experimental Animal Facility, National Institute for Research in Reproductive Health, JM Street, Parel Mumbai, 400 012 India

**Keywords:** Uterus, Endometrium, Stem cells, Regeneration, VSELs, OCT-4

## Abstract

**Background:**

Endometrium undergoes dramatic growth, breakdown and regeneration throughout reproductive period in mammals. Stem cells have been implicated in the process however their origin, nature, anatomical localization and characterization still remain obscure. Classical concept of presence of stem cells in the basal layer of endometrium was recently challenged when side population and label retaining cells were found to be distributed throughout endometrium. We have earlier reported very small embryonic-like stem cells (VSELs) in adult mammalian ovary and testis as a small population of cells with nuclear OCT-4 along with progenitors (spermatogonial stem cells and ovarian germ stem cells) with cytoplasmic OCT-4. Present study was undertaken to gauge presence of VSELs in bilaterally ovariectomized mouse uterus and their modulation by hormones.

**Methods:**

Bilaterally ovariectomized mice were subjected to sequential estradiol and progesterone treatment in order to induce proliferation, differentiation and remodeling (regeneration). Stem cells were studied in tissue smears after H & E staining and after sorting using SCA-1 by immuno-localization and qRT-PCR studies (*Oct-4A, Nanog and Sca-1*). Flow cytometry studies were also undertaken to confirm the presence of VSELs in mouse uterus.

**Results:**

Two distinct populations of stem cells with dark stained nucleus and high nucleo-cytoplasmic ratio were detected in ovariectomized mouse uterus. These cells were sorted using SCA-1 and comprised smaller VSELs with nuclear expression of OCT-4 and slightly bigger, more abundant progenitors termed as endometrial stem cells (EnSCs) with cytoplasmic OCT-4. RT-PCR studies showed presence of pluripotent transcripts (*Oct-4,* Sca-1) and flow cytometry confirmed the presence of 0.069% of LIN-/CD45-/SCA-1+ VSELs. These stem cells were distinctly regulated during endometrial growth, differentiation and regeneration as evidenced by qRT-PCR results.

**Conclusions:**

VSELs are present in normal uterus and also under conditions of atrophy induced by bilateral ovariectomy. Marked increase in EnSCs is associated with endometrial growth and regeneration. Further studies are warranted to define the niche for these stem cells and whether EnSCs arising from the pluripotent VSELs are common progenitors for epithelial and stromal cells or not remains to be addressed. Results of the present study will help in better understanding of endometrial pathologies and their management in the future.

**Electronic supplementary material:**

The online version of this article (doi:10.1186/s13048-015-0138-2) contains supplementary material, which is available to authorized users.

## Introduction

A novel population of pluripotent stem cells termed very small embryonic-like stem cells exists in various adult body organs as shown by seminal contributions made by Ratajczak’s group [1,2]. These cells are believed to be the primordial germ cells or their precursors which while migrating to the gonadal ridges via the dorsal mesentery during early embryonic development, possibly migrate to various developing body organs and persist throughout life as a backup population of pluripotent stem cells to maintain tissue homeostasis [3-5]. VSELs are very small in size (smaller than red blood cells), have a characteristic spherical shape, high nucleo-cytoplasmic ratio, exhibit intensely Hematoxylin stained large nucleus surrounded by a thin rim of cytoplasm, are positive for alkaline phosphatase, can be isolated in mice as LIN-/CD45-/SCA-1+ cells by flow cytometry, express pluripotent transcripts including nuclear Oct-4, Nanog, Sox-2 and cell surface SSEA-1 and have long telomeres. VSELs express pluripotent markers, self-renew and have the potential to differentiate into all three germ layers in mice [6] and humans [7], however; unlike pluripotent embryonic stem cells, they do not form teratoma nor compliment developing embryos. This is due to the novel epigenetic mechanism of imprint erasure on paternally imprinted DMRs (H19-Igf2, RasGRF1) exhibited by VSELs [3,8] as they appear slightly later during embryonic development (in epiblast-stage embryo) compared to ES cells derived *in vitro* from the inner cell mass. ES cells undergo symmetric cell divisions *in vitro,* are immortal in nature, form teratoma and compliment developing embryo in contrast to VSELs which exhibit extreme quiescence *in vitro* and possibly undergo asymmetric cell divisions *in vivo* to self-renew and give rise to progenitors which expand in large numbers and further differentiate into specific cell types depending on their location.

We have reported relatively quiescent, pluripotent VSELs with nuclear OCT-4 in adult mammalian testis and ovary [9,10]. Besides VSELs, there exists another population of tissue specific progenitors derived from the VSELs which are slightly bigger in size, have cytoplasmic OCT-4 and are more active including spermatogonial stem cells (SSCs) in testis and ovarian germ stem cells (OGSCs) in ovary. This existence of two stem cells populations in gonads is in agreement with similar concept of quiescent and active stem cell populations proposed in bone marrow, skin and gut [11,12]. Stem cells are lodged in the ovary surface epithelium and in the testicular seminiferous epithelium. VSELs have remained elusive so far because of their very small size and are not easily visualized in paraffin sections; rather we first detected them in smears prepared after enzymatic digestion of the gonadal tissue. VSELs located in the ovary surface epithelium express gonadotropin (follicle-stimulating hormone, FSH) receptors and undergo self-renewal and germ cell nest formation after FSH treatment [13-16]. Similarly, Kucia et al. [3] reported that bone marrow VSELs express mRNA for several pituitary and gonadal hormone receptors and administration of sex hormones directly stimulates expansion (∼2–3x) of VSELs and HSCs in bone marrow associated with increased BrdU incorporation. Because of their quiescent nature, VSELs survive total body radiation in mouse bone marrow (HSCs are destroyed) [17] and also chemotherapy in mice testes (SSCs, spermatocytes and haploid sperm get destroyed) [18] and ovaries (OGSCs, follicles get destroyed) [19]. On providing a healthy microenvironment (by way of inter-tubular transplantation of healthy Sertoli or mesenchymal cells) resulted in restoration of spermatogenesis in chemoablated testis [18]. Similarly the VSELs in chemoablated ovaries retain potential to initiate neo-oogenesis and germ cells cluster formation [19].

Present study was undertaken to investigate whether similar populations of VSELs and endometrium specific progenitors exist in the mouse uterus and if they do, whether they are modulated by sex hormones. Uterine endometrium is a dynamic tissue in the body which undergoes regular proliferation, differentiation, growth, breakdown and shedding and again regenerates more than 400 times during the reproductive life in humans [20]. After the endometrium is shed as part of the physiologic, normal 28 days menstrual cycle, it regenerates to a thickness of 4-7 mm within 4-10 days [21]. Besides it also undergoes extensive growth during pregnancy to accommodate the growing fetus and following hormone replacement therapy in menopausal women. Stem cells have been implicated in the process of endometrium remodeling, regeneration and also during various disease conditions like endometriosis and endometrial hyperplasia, carcinoma, leiomyomas and adenomyosis [22]. Data is now emerging using various approaches on endometrial stem cells and their possible location however, a clear consensus on their origin, nature, anatomical location and character is still lacking [23].

Bilaterally ovariectomized mice model has been used in the present study as it allows manipulation of endometrial cells in response to the steroids administered and has been used by various investigators in the past [24-26]. This model was recently used as a functional model of endometrial breakdown and repair [27] that mimics events of menstruation in women [24,28,29]. It is intriguing to point out here that pluripotent transcripts have been reported in both mice [30] and human [31-35] endometrium. However, most of the groups have reported cytoplasmic OCT-4 which is not crucial for stemness and has resulted in lot of confusion in the available literature [36,37]. But since cells with cytoplasmic OCT-4 have been reported in the uterus, we speculated that there may also exist a small population of pluripotent stem cells with nuclear OCT-4A which gives rise to the cells with cytoplasmic OCT-4. Our study is a preliminary report and further studies to functionally characterize the stem cells and their ability to differentiate into glands and stromal cells are ongoing.

## Materials and methods

The study was approved by Institute Animal Ethics Committee and was carried out using in-house bred eight-weeks old Swiss mice housed in the Experimental Animal Facility. They were kept in a temperature and humidity controlled room on a 12 hr light/12 hr darkness cycle with free access to food and water.

### Study design

#### Presence of VSELs in mouse uterus

Despite the presence of VSELs in the ovarian [38] and testicular [39] smears, they cannot be easily observed in histological sections due to their very small size. Indeed these stem cells have been described as the ‘lost pearls’ that are smaller than red blood cells in size and easily get missed as debris [40]. Thus similar to our earlier efforts of detecting VSELs in smears and later in sections, whole uterine tissue was used to prepare smears to search for the VSELs since we were sure neither of their presence nor their exact location/niche in uterus. A normal mouse uterus was used to prepare cell suspension by enzymatic digestion. Once the VSELs were detected in the smears and also by flow cytometry as SCA-1 positive cells in the size range of 3-5 μm, the whole study was planned and various experiments were undertaken. Detailed enumeration of VSELs in mouse uterus as LIN-/CD45-/SCA-1+ cells was done at the Stem Cell Institute at James Graham Brown Cancer Center, University of Louisville by PG (refer to Additional file 1 for details).

### Characterization of uterine VSELs

The uterine smears were used to study expression of octamer binding transcription factor (OCT-4) specific for pluripotent state [41] in VSELs. The cell suspension after enzymatic digestion was subjected to immuno-magnetic enrichment of VSELs using SCA-1. Both the positive and negative SCA-1 sorted fractions were subjected to RT-PCR to detect presence of pluripotent transcripts including Oct-4A (specific for nuclear OCT-4A isoform), Sca-1(stem cell antigen-1) and Nanog (homeobox transcription factor). Oct-4 (representing total Oct-4 inclusive of all transcripts of Oct-4) transcript which is suggestive of the presence of VSELs and their immediate descendant ‘progenitors’ was also studied.

### Modulation of VSELs by different hormonal conditions

**Figure 1:**
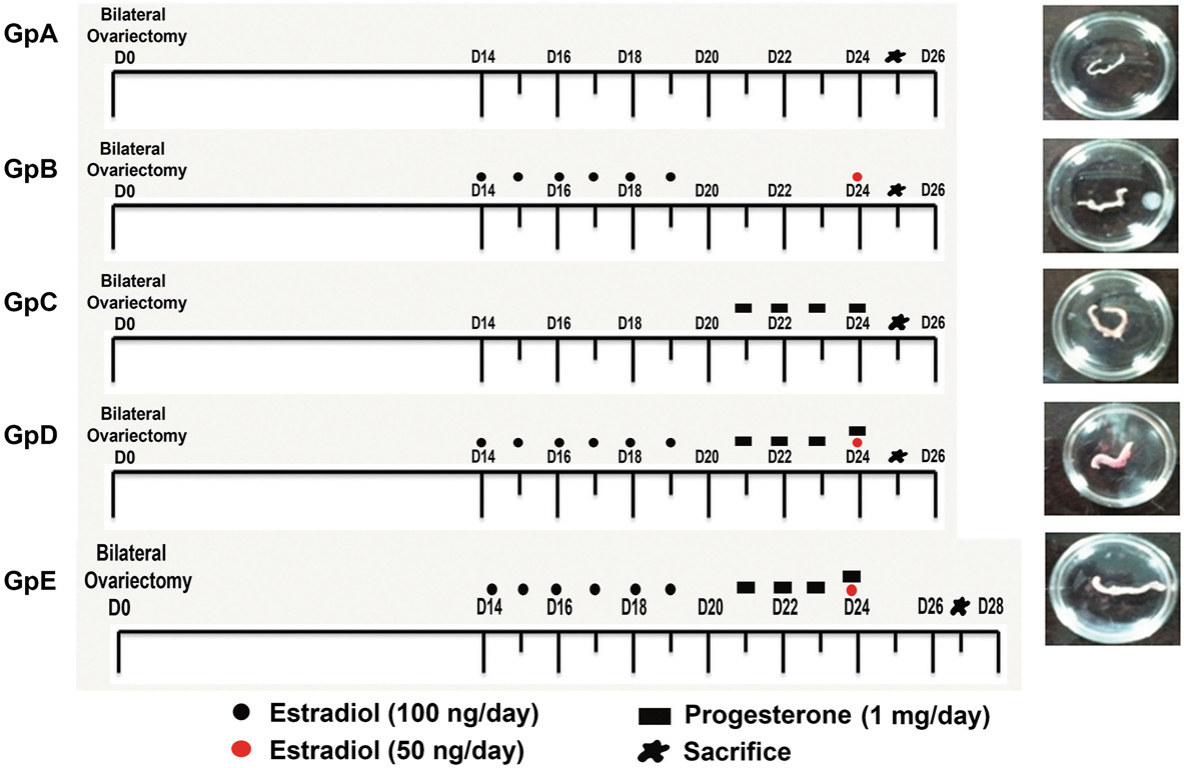
**Treatment schedule and gross appearance of uteri at the time of sacrifice.** Various treatment groups (Gp) were used to study how mouse uterine stem cells are modulated by hormones under varying conditions of endometrium including atrophy, growth and remodeling/regeneration. Mice were bilaterally ovariectomized (on Day 0) and 14 days later were subjected to various hormonal treatments as shown. Gp A received no hormonal treatment, were sacrificed on Day 25 and the uterus appeared atrophied evident by its very thin appearance compared to other groups. Gp B, D & E received estradiol (E, 100 ng/day) priming for six days (D14-19). Gp B was treated with 50 ng/ml estradiol on Day 25 and sacrificed 24 hrs later to study the proliferative effect of estradiol on endometrial cells. Gp C was not primed with estradiol, only received progesterone (P, 1 mg/day) on Days 21-24 and sacrificed 24 hrs later to study the effect of progesterone alone on the endometrium. Gp D received estradiol priming, progesterone on Days 21-24 and also estradiol (50 ng) on Day 24, and was sacrificed 24 hrs after the treatment on Day 25 was the E + P treated receptive phase endometrium with maximal growth. Gp E reflected condition of remodeling/regeneration resulting by withdrawal of hormones. These mice were treated as Gp D and were sacrificed 48 hrs after Gp D i.e. on Day 27.

To study whether VSELs are modulated by different hormonal conditions during endometrial atrophy, proliferation, differentiation and remodeling, the mice were subjected to bilateral ovariectomy under aseptic conditions. Fourteen days after bilateral ovariectomy, mice were divided into 5 groups (Figure 1) including (i) control OVX group which received only sesame oil injections (ii) estradiol (E) (iii) progesterone (P) (iv) E + P (v) 48 hrs after withdrawal of E + P. These different groups represented uterine atrophy (Gp A); proliferation (expected after E and not after P treatment, Gp B & C); secretory phase (E + P group, Gp D) and remodeling (48 hrs of withdrawal of E + P, Gp E). Various events like uterine proliferation and apoptosis was confirmed by qRT-PCR using specific markers. Proliferating cell nuclear antigen (Pcna) was used as a marker for proliferation. It is a key cell-cycle regulator and has an important role to play during DNA repair and replication. Ratio of Bcl-2/Bax was used to confirm apoptosis. Bcl-2 is a proto-oncogene and Bax is a pro-apoptotic protein that induces apoptosis by homodimerization and heterodimerization with Bcl-2. VSELs specific transcripts (Oct4, Oct4A, Nanog, Sca-1) were also studied by qRT-PCR in various treatment groups to evaluate whether they are differentially modulated under different conditions.

## Methods

### Bilateral ovariectomy and hormone treatment

Bilateral ovariectomy was performed by expert veterinarians (SM and DM) using standard protocols. Beta-estradiol (E, Sigma-Aldrich, Germany) stock for injections was prepared by dissolving 1 mg estradiol in 100 μl of ethanol and making up to 1 ml with sesame oil. 10 mg progesterone (P, Sigma) was dissolved in 100 μl of chloroform and final volume made to 1 ml by adding 900 μl of sesame oil. Hormones were injected subcutaneously in a volume of 100 μl. Two weeks after bilateral ovariectomy, the mice were subjected to various treatment schedules as shown in Figure 1. Mice in Gp A received only sesame oil injections and sacrificed on day 25 whereas Gp B, D & E were initially primed by daily estradiol (100 ng/day) injections for 6 days (Day 14-19 after ovariectomy). Mice in Gp B were injected estradiol (50 ng) on day 24 and sacrificed 24 hrs later on day 25. Similarly mice in GpC were injected progesterone (1 mg/Kg) on days 21-24 and sacrificed 24 hrs later on day 25. Gp D was initially primed with estradiol and then injected progesterone on days 21-24 along with estradiol (50 ng/day) on day 24 and then sacrificed on day 25. Mice in group E received similar treatment as Gp D and were sacrificed on day 27, 48 hrs after Gp D. Estradiol priming for six days was essential as it is well known that progesterone alone is incapable of expressing its own receptors [42]. The tissue collected after various treatments was processed appropriately to make uterine tissue cell smears, for histology flow cytometry and RNA extraction. Formalin fixed uterine tissue was processed and embedded in paraffin using standard protocols. 5 μm thick sections were prepared and stained with Hematoxylin and Eosin for studying the histo- architecture of uterus after different hormonal administration. The representative areas were photographed and data recorded.

### Smears preparation from uterine tissue

Briefly the uterine tissue was minced into small pieces and then incubated with collagenase (1 mg/ml) & Trypsin (1 mg/ml) and DNAse (0.5 mg/ml) in DMEM culture media at 37°C for 30 minutes. After filtering through 40 μm filter, the filtrate was used to make smears. After air drying, the smears were fixed in 4% PFA, washed 2-3 times with phosphate buffer saline (PBS), air dried and stored at 4°C till further use. These smears were stained with Hematoxylin and Eosin using standard method. The smears were also stained with 4',6-diamidino-2-phenylindole, dihydrochloride (DAPI) for few seconds to demonstrate the presence of VSELs, as we know from our experience on gonadal stem cells that VSELs (with abundant euchromatin) do not stain easily with DAPI compared to the progenitors [38] because of the presence of abundant open euchromatin and DAPI selectively stains specifically to A-T base pairs, producing a much stronger signal on the heterochromatin than on the euchromatin [43,44].

### Enrichment of SCA-1 positive cells

Single cell suspension was prepared from uterine tissue as described above. SCA-1 positive cells were immuno-magnetically enriched using FITC tagged SCA-1 antibody and Easysep kit (Stem Cell Technology, Canada) according to manufacturer’s instructions. Briefly 10^8^ cells were immuno-stained with SCA-1 antibody (BD Life Sciences, USA) for 15 minutes at room temperature (RT), followed by incubation with FITC selection cocktail for 15 minutes. The cell suspension was then incubated with magnetic nanoparticles for 10 minutes at RT and placed on a magnetic particle concentrator. After isolation, enriched SCA-1^+^ cells fraction as well as the negative fraction were used for making smears and for RNA extraction.

### Immuno-staining of OCT-4 on smears

The cells smears were washed with PBS and then incubated for 30 min in 3% hydrogen peroxide followed by antigen retrieval in boiling sodium citrate saline buffer of pH 6 for 5 min. After cooling, the smears were washed with PBS for 5 min and then blocked with 10% normal goat serum in PBS. After removing excess blocking reagent, the slides were incubated with primary antibody (polyclonal OCT4 from Millipore) for two hours at room temperature. The detection was done using anti-rabbit Vecta ABC kit (Vector Laboratories, USA) according to manufacturer’s instructions. Color development was done using 3,3' diaminobenzidine (DAB) (Biogenex, USA). After counterstaining with Hematoxylin, the slides were observed under Nikon 90i microscope. Representative areas were photographed. Total protein isolated from atrophied uterus was subjected to Western blotting for OCT-4 (Additional file 1: Figure S2).

### Immuno-fluorescence and confocal microscopy

SCA-1 sorted cells were used for studying expression of OCT-4 by immuno-fluorescence. For this, following washes with 0.5% BSA solution, the cells were permeabilized using 0.3% Triton-X 100 for 5 minutes. Blocking was done with 3% BSA solution for 1 hour. Smears were incubated with polyclonal OCT-4 antibody which stains both the isoforms of OCT-4 (Abcam) at 4°C overnight. Next day the smears were washed with wash buffer and incubated with fluorescent tagged secondary alexafluor-488 antibody for 2 hours at RT in dark. The slides were then washed thrice with PBS and counter-stained with DAPI for 15 minutes. Images were captured by laser scanning confocal microscope (Carl Zeiss, Germany).

### RT-PCR and qRT-PCR studies

Details of primers used in the present study are shown in Table 1.

**Table 1:** **List of primer sequence used for RT-PCR and qRT-PCR**

**Gene name**	**Primer sequence**	**Annealing temperature (°C)**	**Amplicon size (bp)**
Pcna	F:GATGCCGTCGGGTGAATTTG	55	182
	R:TCTCTATGGTTACCGCCTCCT		
Bax	F:GTTTCATCCAGGATCGAGCAG	55	488
	R:CATCTTCTTCCAGATGGTGA		
Bcl-2	F:CCTGTGGATGACTGAGTACC	55	128
	R:GAGACAGCCAGGAGAAATCA		
Oct-4	F: CCTGGGCGTTCTCTTTGGAAAGGTG	66	198
	R: GCCTGCACCAGGGTCTCCGA		
Oct-4A	F: CCATGTCCGCCCGCATACGA	61	235
	R: GGGCTTTCATGTCCTGGGACTCCT		
Nanog	F: CAGGAGTTTGAGGGTAGCTC	61	223
	R: CGGTTCATCATGGTACAGTC		
Sca-1	F: AGAGGAAGTTTTATCTGTGCAGCCC	66	276
	R:TCCACAATAACTGCTGCCTCCTGA		
18S	F:GGAGAGGGAGCCTGAGAAAC	61	198
	R: CCTCCAATGGATCCTCGTTA		
GAPDH	F: GTCCCGTAGACAAAATGGTGA	58	435
	R: TGCATTGCTGACAATCTTGAG		

### RNA isolation and cDNA synthesis

The uterine tissue was collected in Trizol (Invitrogen, Carlsbad, CA, USA) for RNA extraction by standard protocol and then treated with DNase I (Amersham Biosciences, Piscataway, NJ) at 37°C for 30 min to remove any genomic DNA contamination. First-strand cDNA was synthesized using the cDNA synthesis Kit (Bio-Rad, USA) according to the manufacturer’s instructions. Briefly, 1-2 μg of total RNA was incubated with 5X reaction mix and reverse transcriptase mix. The reaction was carried out in G-STORM thermocycler (Gene Technologies, Braintree, UK). The reaction mix was first incubated at 25°C for 5 min, then at 42°C for 30 min and finally at 85°C for 5 min. for cDNA synthesis.

*RT-PCR for pluripotent stem cells specific markers:* In order to demonstrate the presence of VSELs by RT-PCR, various transcripts (Oct4, Oct4A, Nanog, Sca1) were amplified using a temperature gradient to establish the optimal annealing temperature which varies from tissue to tissue. Briefly, the cDNA mix (2 μl) was amplified using 0.2 mM of each primer as described (Table 1), 1.25 unit of DreamTaq DNA polymerase (Fermentas) in 1x Dream Taq buffer (Fermentas) and 0.2 mM dNTPs in a G-STORM thermocycler. Amplification was carried out for 35 cycles, with each cycle consisting of denaturation at 94°C for 30 sec, annealing at the specified temperature for each set of primers (Table 1) for 20 sec, and extension at 72°C for 30 sec. The products were analyzed on 2% agarose gel stained with 0.5 μg/ml ethidium bromide (Bangalore Genei, India). The product size was approximated using a 100-bp DNA ladder (Bangalore Genei). The negative control did not include cDNA in the reaction mixture and cDNA from mouse ovary tissue was used as positive control.

*Quantitative RT- PCR (qRT-PCR):* Later, using the standardized annealing temperature, qRT-PCR was carried out to study how these transcripts are regulated by steroid treatment. The expression levels of these gene transcripts in relation to housekeeping gene transcript 18S were estimated by CFX96 real-time PCR system (Bio-Rad Laboratories, USA) using SYBR Green chemistry (Bio-Rad). The amplification conditions included initial denaturation at 94°C for 3 min followed by 40 cycles comprising of denaturation at 94°C for 30 seconds, primer annealing for 20 sec and extension at 72°C for 30 sec. The final step included incubation at 94°C for 20 s to remove any secondary structures. The fluorescence emitted at each cycle was collected during the extension step of each cycle. The homogeneity of the PCR amplicons was verified by running the products on 2% agarose gels and also by studying the melt curve. All PCR amplifications were carried out in duplicate. Mean Ct values generated in each experiment using the CFX Manager software (Bio-Rad) were used to calculate the mRNA expression levels. Since ΔCt is inversely proportional to relative mRNA expression levels, the levels were calculated manually by the ΔCt method.

## Results

### Presence of VSELs in mouse uterus

**Figure 2:**
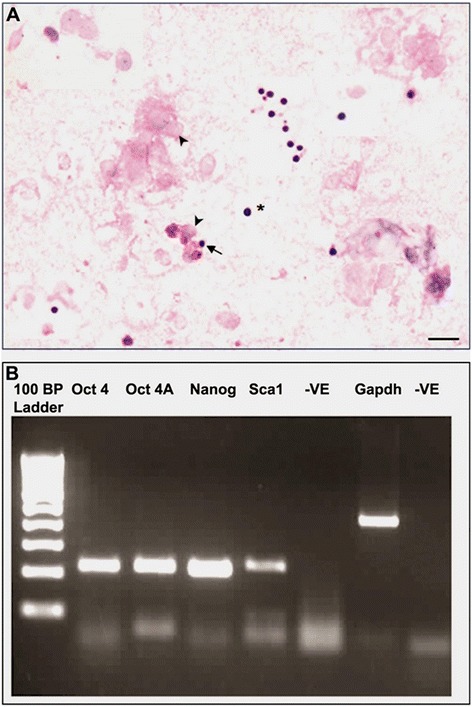
**Detection of VSELs in mouse uterus.** Panel **A** Hematoxylin and Eosin stained uterine smears prepared after enzymatic digestion of the tissue. Note the presence of distinct spherical stem cells with dark stained nucleus with high nucleo-cytoplasmic ratio. In contrast, the somatic uterine cells have pale stained nucleus surrounded by abundant cytoplasm. Please note that 5-6 different fields have been combined to make this figure. Scale bar represent 20 μm. Lower Panel **B** shows RT-PCR results for pluripotent markers *Oct 4A, Nanog, Sca-1, Oct 4* and *Gapdh* in bilaterally ovariectomized uterus (Gp A). Results show that pluripotent stem cells exist in atrophied endometrium.

H & E stained smears of uterine cell suspension clearly showed the presence of very small sized cells with dark Haematoxylin stained nucleus surrounded by a thin rim of cytoplasm (Figure 2A). These cells were distinct from the somatic cells with pale stained nucleus surrounded by abundant cytoplasm. The stem cells appear similar to the VSELs reported by our group in testis and ovary (Bhartiya et al., [9]). Flow cytometry studies confirmed the presence of 0.0685% of LIN-/CD45-/SCA-1+ VSELs in mouse uterus (Additional file 1: Figure S1). RT-PCR analysis (Figure 2B) confirmed the presence of pluripotent transcripts of expected size for *Oct4, Oct4A, Sca1 and Nanog* in the ovariectomized uterine tissue (Gp A).

### Characterization of uterine VSELs

**Figure 3:**
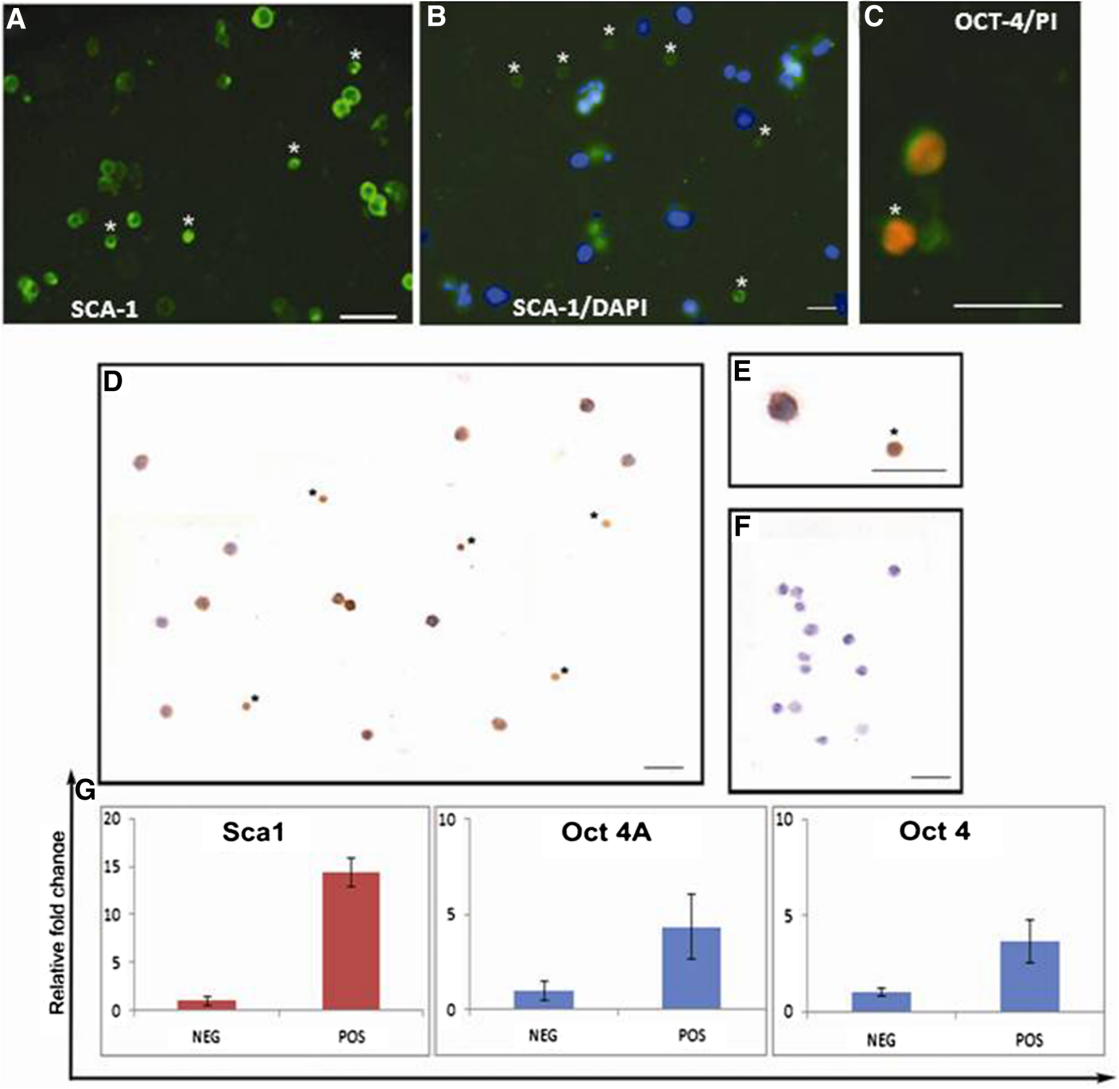
**Characterization of uterine stem cells.** **A**. SCA-1 sorted cells under microscope appear green and are of two distinct sizes including small sized cells which possibly are the very small embryonic-like stem cells VSELs (asterix) and slightly bigger endometrial stem cells EnSCs. **B**. DAPI stained smear shows that the somatic cells negative for SCA-1 stain nicely with DAPI whereas the stem cells do not stain with DAPI. Similar data was obtained for ovarian stem cells^33^; these cells do not stain with DAPI within 2-3 secs possibly because being pluripotent they have open chromatin. **C**. Higher magnification of OCT-4 stained cells with propidium iodide used as a counterstain shows cells have distinct nuclear staining of OCT-4. **D**. OCT-4 immuno-localization on the smears. Note the presence of two distinct sized cells which stain positive for OCT-4 including the VSELs (asterix) and slightly bigger EnSCs. **E**. Nuclear versus cytoplasmic OCT-4 staining can be clearly observed at higher magnification. **F**. Negative control. Scale bars represent 20 μm. Lower panel **G** shows the qRT-PCR results of negative (NEG) and positive (POS) fractions after immuno-magnetic sorting of SCA-1 positive cells. As evident the stem cell markers *Sca1, Oct 4A and Oct 4* are highly enriched in the positive fraction.

Uterine cell suspension obtained by enzymatic digestion was used to enrich SCA-1 positive VSELs. SCA-1 sorted cells under fluorescent microscope showed very distinct surface staining for SCA-1 (Figure 3A) and did not stain with DAPI (Figure 3B) compared to somatic cells which were stained with DAPI within 2 secs. These results are similar to our earlier results where ovarian VSELs also poorly stain with DAPI [38]. Immuno-localization for OCT-4 on sorted stem cells showed the presence of two distinct cell types expressing OCT-4 including few smaller cells with nuclear OCT-4 and large number of cells with cytoplasmic OCT-4 (Figure 3C-E). qRT-PCR analysis showed that the pluripotent transcripts (Sca-1, Oct-4A and Oct-4) were enriched in the immuno-magnetically positively sorted (POS) compared to the negative (NEG) fraction suggesting that pluripotent VSELs do exist in the uterus and can be sorted using SCA-1 (Figure 3, bottom panel).

Thus above results showed the presence of LIN-/CD45-/SCA + VSELs ranging in size from 3-5 μm with nuclear OCT-4 and expressing Oct-4, Nanog and Sca-1 mRNA transcripts in mouse uterus. Two distinct sizes of stem cells with nuclear OCT-4 in smaller VSELs and slightly bigger cells with cytoplasmic OCT-4 were visualized. The results are similar to the VSELs reported in testis and ovary by our group. Interestingly the bigger ‘progenitors’ (immediate descendants of VSELs) with cytoplasmic OCT-4 are the spermatogonial stem cells (SSCs) in testis, ovarian stem cells (OSCs/OGSCs) in ovary and we term them as endometrial stem cells (EnSCs) in the endometrium. Since we have observed EnSCs in smears, their true shape and localization remains to be studied in the endometrium. They could possibly be the cytoplasmic OCT-4 positive mesenchymal cells described by several groups in the endometrium [45,46].

### Modulation of VSELs by hormones

**Figure 4:**
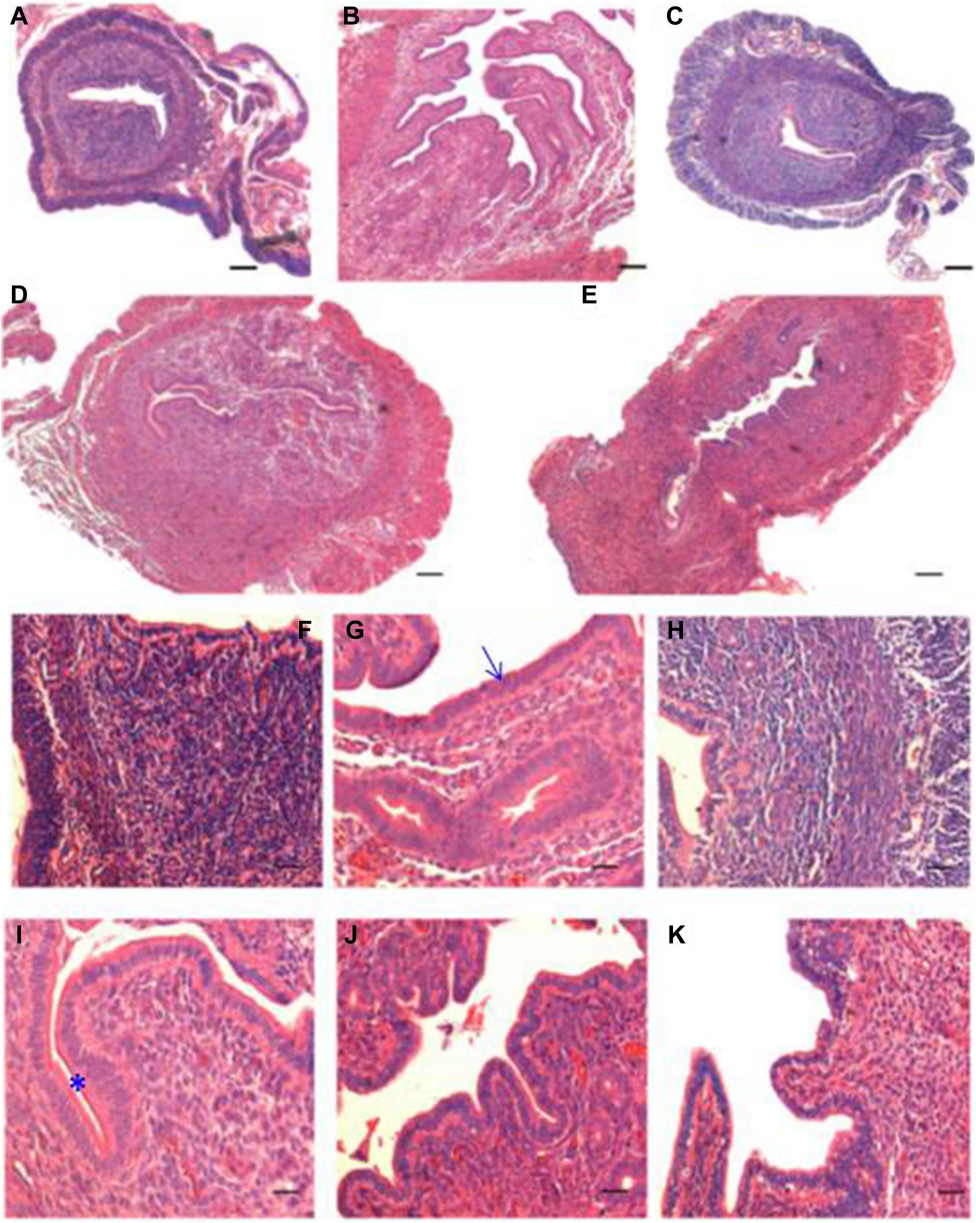
**Histology of mouse uterus of different treatment groups. A** and **F** Ovariectomized GpA. **B** and **G** Estradiol alone GpB. **C** and **H** Progesterone alone GpC **D** and **I** E + P GpD **E**, **J** and **K** E + P withdrawal GpE. As evident the ovariectomized uterus was atrophied **(A)** and the cells were tightly packed **(F)** whereas estradiol treatment resulted in increased size of the uterus **(B)** with increased proliferation in both the glands and stromal cells **(G)**. Epithelial cells were tall and columnar and frequent mitotic figures were observed. Luminal epithelium appeared highly folded, glands were also bigger and lot of edema in the stromal compartment. Progesterone treatment exerted minimal effect and the uterus remained small in size **(C)** with densely packed cells **(H)**. E + P treatment resulted in a receptive endometrium with characteristic narrowing of uterine lumen **(D)**, tall epithelial cells lined the lumen and the glands and lot of edema and increased vascularity was evident in the stromal compartment. In contrast to the apically placed nuclei after estradiol treatment **(G)**, in E + P treated section the nuclei are more centrally placed **(I)**, suggestive of a receptive condition when nutrients required for successful implantation shift to the apical end of the epithelial cells. Also secretory activity was evident on the apical surface of luminal epithelial cells. Withdrawal of E + P resulted in repair and remodeling suggested by the presence of a compacted uterus **(E)**, small epithelial cells with increased nucleo-cytoplasmic ratio and dense compact stromal cells **(J & K)**. Scale bars represent 20 μm.

**Figure 5:**
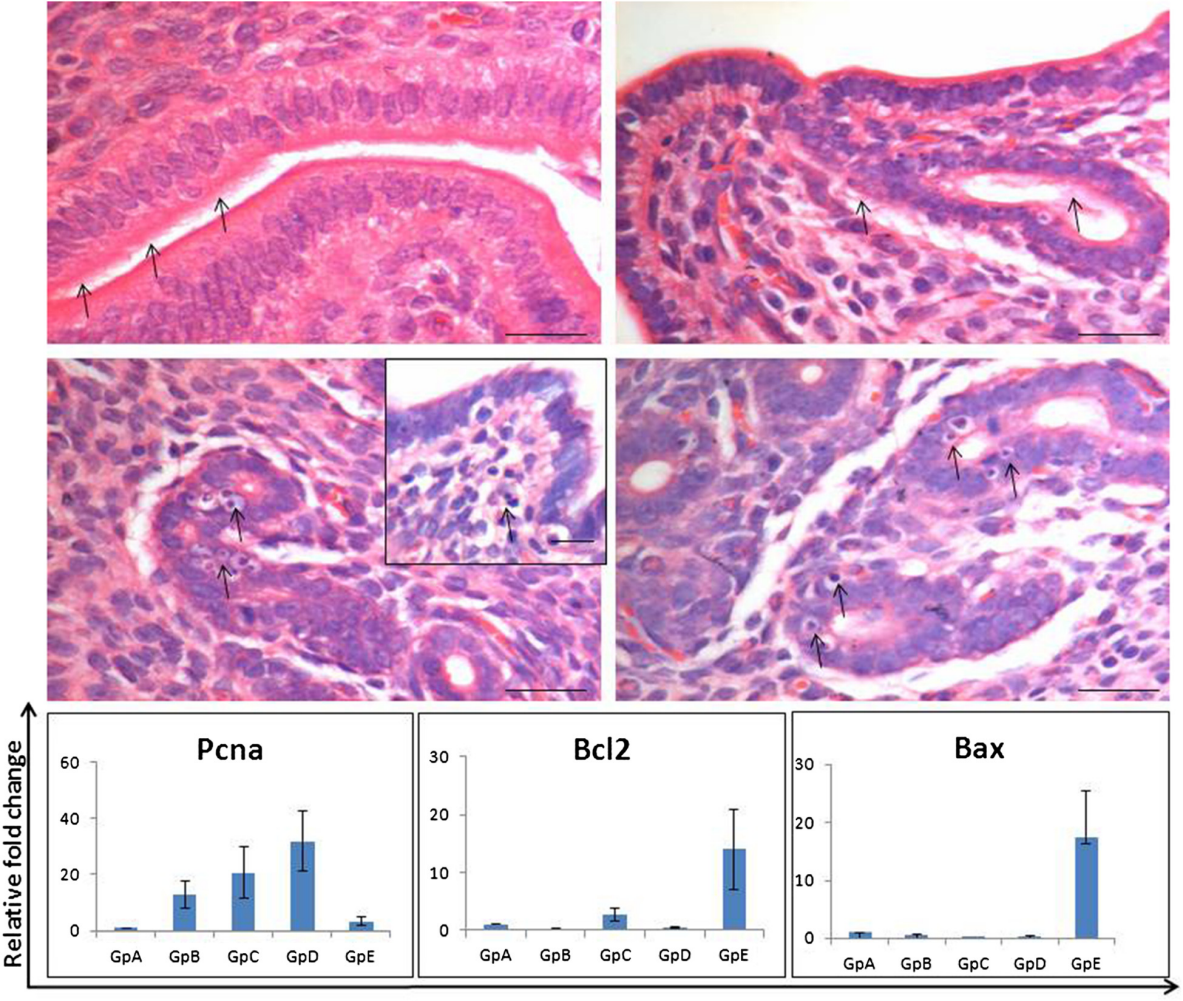
**Higher magnification of uterine sections of Gp D and E.** Luminal epithelium in Gp D (A) comprised tall, columnar cells with centrally placed nuclei. Note the presence of secretory activity in the lumen. Withdrawal of E + P (C-D) resulted in reduction in epithelial cell height with increased nucleo-cytoplasmic ratio and the nuclei were apically placed. The nuclei appeared more apical and signs for remodeling and apoptotic bodies were evident in luminal and glandular epithelium and also in the stromal compartment. Scale bar represent 20 μm. Bottom panel shows the qRT-PCR results of specific transcripts reflecting proliferation (Pcna) and apoptosis (Bax and Bcl2). As evident Pcna was minimally expressed in Gp A and E whereas Bax and Bcl2 were unregulated in Gp E suggestive of increased apoptosis, in agreement with the histology data.

*Effect of steroid treatment on mouse uterus*: As evident, the uterus responded well to the treatment, being thin and atrophied after bilateral ovariectomy and showed variable morphology after various treatments (Figure 1). Histology studies (Figures 4 & 5) showed expected changes in the uterine histology with maximum atrophy (evident by the size in cross-section) observed post ovariectomy (Gp A). The uterus showed growth and extensive proliferation after estradiol treatment (Gp B) compared to progesterone alone (Gp C). Combined estradiol and progesterone (E + P) resulted in differentiation and a receptive endometrium (Gp D) with characteristic narrowing of lumen and secretory activity in the lumen. Withdrawal of E + P for 48 hrs (after mice in Gp D were sacrificed) resulted in extensive remodeling (Gp E) associated with reduction in epithelial cell height (Figure 4). At higher magnification (Figure 5) luminal epithelium showed characteristic features of a receptive stage in Gp D comprising of tall epithelial cells with oval shaped nuclei in the middle, sub-nuclear vacuolation and secretory activity at the luminal surface. Whereas in Gp E the luminal epithelial cells reduced in size due to withdrawal of hormones resulting in increased nucleo-cytoplasmic ratio. Increased vacuolation and apoptotic bodies (in luminal and glandular epithelium as well as in the stromal compartment) could be observed at places, suggestive of remodeling, repair and regeneration (Figure 5B-E). The effect of steroid treatment at transcript level (Figure 5 Lower panel) confirmed the histological findings. Pcna (marker suggestive of DNA synthesis and proliferation) showed minimal expression in Gps A and E (after ovariectomy and during hormone withdrawal). Whereas it was variably expressed in groups B-D. Bcl-2 was also variably expressed in all groups whereas Bax transcripts were observed only in Gp E. The ratio of Bax and Bcl-2 (suggestive of apoptosis) were increased in Gp E only confirming that withdrawal of E + P for 48 hrs resulted in remodeling of the endometrium and involved apoptosis. Thus we successfully obtained a mouse model to study how VSELs are modulated in an atrophied endometrium (Gp A), proliferative endometrium (Gp B compared to Gp C), secretory endometrium (Gp D) and endometrium which is undergoing remodeling and regeneration (Gp E).

Quantitative RT-PCR studies for pluripotent markers Oct-4A, Nanog and Sca-1 revealed an interesting pattern. These transcripts were detected in all the groups however; their expression was maximum in Gp A and E. Also Oct-4 (suggestive of the presence of ‘progenitors’ EnSCs) was maximally expressed in the Gp D. It was interesting to note that the ratio of Oct-4 to Oct-4A was equal in the ovariectomized samples suggesting that stem cells in ovariectomized sample mainly comprised of VSELs. In contrast Oct-4 was more than ten-fold increase in Gp D compared to a two-fold increase in Oct-4A, suggesting a higher proportion of progenitors with cytoplasmic OCT-4 when the endometrium undergoes maximum growth/differentiation and gets ready for implantation to occur.

## Discussion

Different strategies have been employed by various investigators to detect endometrial stem cells as shown in Table 2 but a clear picture of the true identity of endometrial stem cells is yet to emerge. Present study for the first time shows the presence of LIN^-^CD45^-^SCA^+^ very small embryonic-like stem cells (VSELs) in the mouse uterus. VSELs (comprising 0.07% of the total cell population as judged by flow cytometry) were observed in uterine cell smears and could be enriched by immuno-magnetic sorting using a cell surface antigen SCA-1. Uterine VSELs are small in size (3-5 μm), spherical in shape, have high nucleo-cytoplasmic ratio, stain intensely with Hematoxylin but poorly with DAPI and express nuclear OCT-4, cell surface SCA-1 and pluripotent transcripts including *Oct4A, Sca1* and *Nanog*. Similar VSELs exist in various adult mouse organs [1], human testes [39], ovaries [38], bone marrow [2,47] and cord blood [48,49]. We succeeded to detect VSELs in uterine smears but the exact location/niche of these cells in the uterus still remains elusive because of their very small size and for the same reason they have evaded the reproductive biologists till date.

**Table 2:** **Various approaches utilized to detect and study endometrial stem cells**

**Various approaches**	**Salient observations and relevant references**
Classic concept	Stem cells may reside in the basal region of endometrium which continuously repairs the functional layer [27,50]
Side population (SP) analysis	Side population cells are distributed throughout endometrium [51-53]
Label retaining cells (LRCs)	Mouse LRCs decline with age, exist in lower region of stroma and exhibit undifferentiated markers including OCT-4 [25]
	LRCs detected in luminal epithelium and stromal cells adjacent to the luminal epithelium [54] in mouse endometrium
	LRCs detected only in the glandular epithelium [22] in humans
Clonogenic assays	Human endometrial stromal progenitor cells can be cultured for more than 15 passages and show high clonogenic efficiency (15%) [55]
	Single endometrial stromal cell can form cellular colonies that can be further serially cloned and differentiated into various mesodermal lineages [56]
Other relevant results	Pluripotent markers have also been reported in human endometrial and endometriotic samples [27-30]
	Bone marrow cells may be implicated in endometrial repair [47,57]

Besides the VSELs with nuclear OCT-4, we also detected slightly bigger cells with cytoplasmic OCT-4 which we term as the endometrial stem cells (EnSCs). These are most likely the immediate multipotent descendants or ‘progenitors’ which arise by differentiation of pluripotent VSELs and are expected to be multipotent and give rise to various endometrial cell types. Differential OCT-4 expression pattern (obtained using a polyclonal antibody which cross-reacts with both OCT-4A and OCT-4B isoforms) has been very useful to differentiate between pluripotent VSELs, the immediate descendants with cytoplasmic OCT-4 and the differentiated progeny which show loss of OCT-4. It is intriguing that similar VSELs exist in ovary, testis, bone marrow and uterus but the immediate descendants are distinct and give rise to different cell types based on where the VSELs are located; spermatogonial stem cells (SSCs) in testis differentiate to form the haploid sperm, ovarian stem cells (OSCs) differentiate into haploid oocytes, hematopoietic (HSCs) and mesenchymal (MSCs) stem cells give rise to various kind of blood cells and similarly the uterine stem cells (EnSCs) are expected to differentiate into various endometrial cell types. Results suggest that the microenvironment/niche where the VSELs are located is crucial and dictates the fate of stem cells. We recently reported that in mouse pancreas after partial pancreatectomy – VSELs give rise to OCT-4 & PDX-1 co-expressing progenitors which then differentiate and give rise to both the beta and acinar cells [58]. Ability of VSELs to give rise to SSCs, OSCs, HSCs, MSCs, EnSCs and pancreatic progenitors reflects their pluripotent state. Ability of VSELs to give rise to three germ layers has already been reported in mice [6] and humans [7]. Further detailed studies are required to understand stem cells-niche interaction as recently suggested [59,60].

Two stem cell populations comprising of a quiescent and actively dividing stem cells have been reported in adult somatic tissues including gut epithelium, hair follicle, bone marrow etc. [11,12]. Similarly we have found that VSELs are the quiescent stem cells in the testis and ovaries whereas the SSCs and OGSCs divide rapidly and undergo clonal expansion prior to initiating differentiation [9,10]. That VSELs comprise the quiescent population of stem cells gets further credence from the reports showing that they survive chemotherapy in mice testis [18], ovary [19] and total body irradiation in bone marrow [17] whereas the actively dividing progenitors are destroyed. These findings are intriguing and suggest that VSELs could be the quiescent stem cell population in various adult organs. VSELs have also been implicated in cancers [61] and are possibly the elusive cancer-initiating cells responsible for recurrence (as they are likely to be spared of oncotherapy because of their quiescent nature). Further studies are required to investigate this.

Cells with cytoplasmic OCT-4 and other pluripotent transcripts have been reported by various investigators in mouse as well as human endometrial and endometriotic tissue [32-35]. There exists lot of confusion because these studies have failed to distinguish and appreciate the relevance of cytoplasmic versus nuclear OCT-4 staining [36,37]. The small sub-population of VSELs with nuclear OCT-4 remained undetected in the mouse uterus till now possibly because they exist in very few numbers, are of very small size and also because VSELs tend to get lost during processing as suggested earlier while studying cord blood and bone marrow stem cells [48]. Mesenchymal stem cells have been reported in the endometrium [45,46]. It has been suggested that the MSCs (negative for CD45, CD34 and positive for CD146, PDGF-receptor-Beta, CD29, CD44, CDE73, CD90 and CD105) get further differentiated into stromal and glandular cells. Evidence to support that VSELs may give rise to the MSCs has been elegantly demonstrated by Taichman et al. [62] wherein they reported that Sca^+^Lin^-^CD45^-^ cells could differentiate into multiple mesenchymal lineages in their study model. Similarly in umbilical cord tissue we have earlier reported that VSELs (with nuclear OCT-4) and MSCs (with cytoplasmic OCT-4) co-exist in the Wharton’s jelly [48]. Existence of two stem cell populations has also been postulated in the endometrium [54] and the endometrial stromal cells are considered lineage cells of endometrial mesenchymal stem cells.

**Figure 6:**
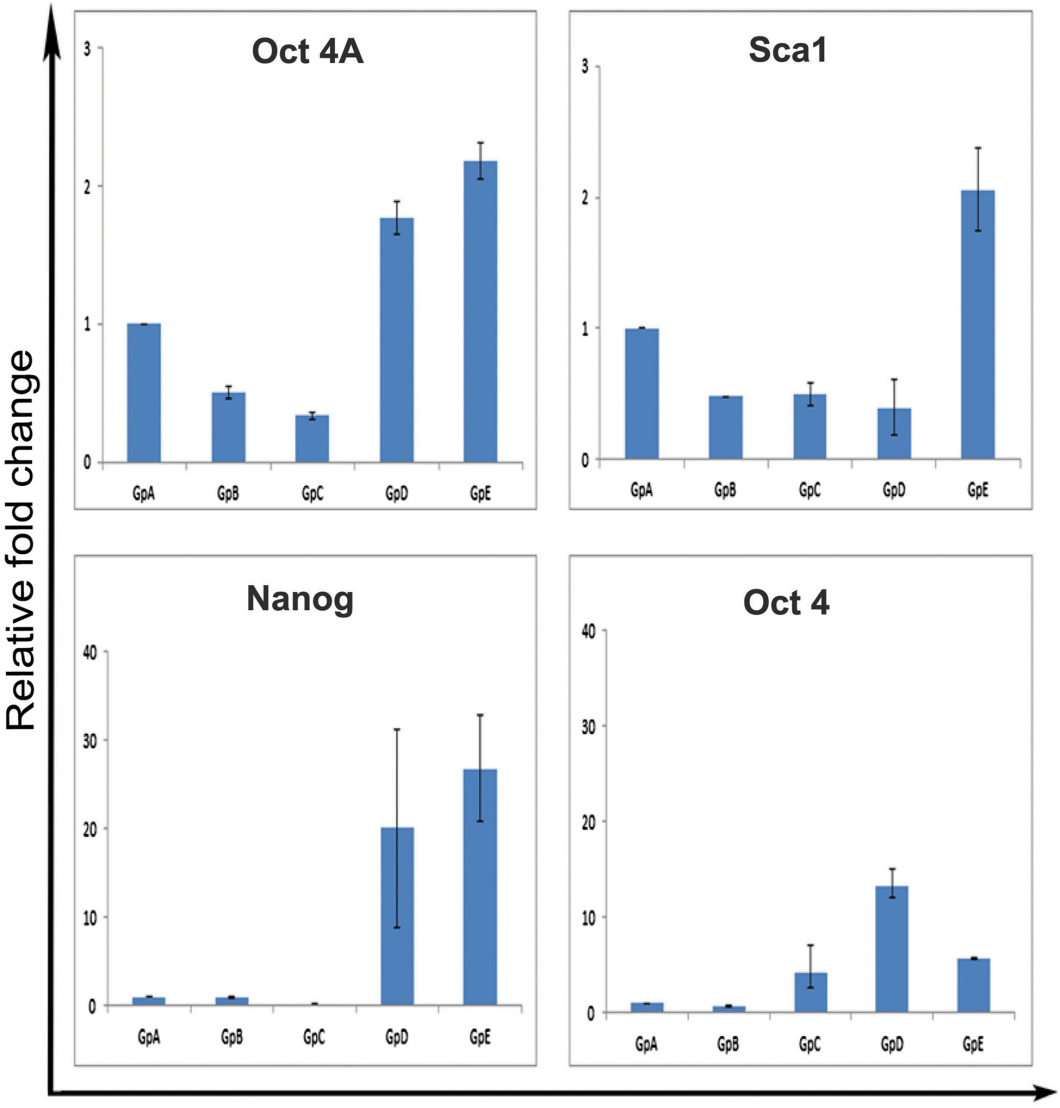
**qRT-PCR results for pluripotent transcripts showing variable effects of hormone treatment.** Oct-4A, Nanog, Sca-1 (pluripotent markers for VSELs) and Oct-4 (all Oct-4 transcripts including Oct-4B indicating presence of EnSCs) in various treatment groups. The pluripotent transcripts are observed in all the treated groups suggesting presence of stem cells in all the conditions, but are modulated by steroid treatment. They are up-regulated in GpA, compared to Gp B and C and are maximally expressed in Gp D and E. Oct-4 is more than 10 fold increased in GpD suggesting presence of EnSCs. Note the scale of Y-axis to appreciate differences between groups. Results suggest that stem cells are present in atrophic endometrium and stem cells activity is greatly increased in E + P group when maximum growth is observed and remain elevated in GpE associated with remodeling and repair. Ratio of Oct-4 to Oct-4A is almost 1 in GpA suggesting most of stem cells are the VSELs whereas in GpD the ratio is greatly increased suggesting presence of large number of progenitors. Results are compilation of more than three biological samples and error bars represent standard error.

Results of the present study further show that uterine VSELs and the progenitors EnSCs are implicated during endometrial atrophy, growth and remodeling/regeneration. Presence of VSELs in ovariectomized atrophied uterus is in agreement with earlier report that a pool of stem cells remains preserved even in atrophic endometrium [63]. Oct-4A and Oct-4 transcripts were equally expressed in Gp A (Figure 6) suggesting that stem cell population comprise mostly of VSELs in the atrophied uterus, however they are unable to differentiate possibly because of a compromised microenvironment/niche, a situation similar to presence of VSELs in chemoablated testis and ovary [18,19]. VSELs and EnSCs are present in the uterus in the E and P treated groups but their numbers are greatly increased in E + P and the withdrawal group. Total Oct-4 transcript is 5-15 fold increase compared to Oct-4A suggesting rapid expansion of progenitors (EnSCs) under these conditions. VSELs give rise to EnSCs which multiply in large numbers and further differentiate into various cell types to bring about growth (Gp D) and remodeling/regeneration (Gp E) of endometrium. In the past, stem cells have been implicated in the process of endometrium remodeling, regeneration and also during various disease conditions like endometriosis and endometrial hyperplasia, carcinoma, leiomyomas and adenomyosis [22]. Thus it becomes pertinent to use a more quantitative approach like flow cytometry and other cellular and molecular techniques to evaluate VSELs and EnSCs in the endometrial tissue from normal and various pathological conditions. Similar to modulation of endometrial VSELs by steroid hormones, VSELs in adult mammalian ovaries are modulated by follicle stimulating hormone [13-16].

Several studies [64,65] suggest that stem cells (isolated as side population SP cells which efflux Hoechst 33342 and also express CD133) from normal endometrium may be involved in the development of endometrial cancer. Kato et al. [66] provided first direct evidence to support that SP cells contribute to endometrial carcinogenesis and are associated with elevated OCT-4 expression. Endometrial hyperplasia (considered as a precursor to endometrial cancer) was observed in mice after neonatal exposure to estradiol and VSELs altered biology was implicated [67]. Thus it is likely that the VSELs which exist in normal endometrium are possibly the embryonic remnants responsible for initiation of endometrial cancer as has been proposed earlier [61].

Engraftment of donor cells in the endometrium (in both glands and stroma) has been reported in women undergoing single-antigen, HLA-mismatched, bone marrow transplantation and also in mice [57,68,69]. Nagori et al. [57] reported beneficial effect of autologus bone marrow cell transplantation in case of Asherman syndrome. Gargett and Healy [70] suggested that main mechanism resulting in beneficial effect of bone marrow cells demonstrated by Nagori’s group, could possibly be to improve the niche rather than true regeneration. Resident stem cells may play a major role in regeneration rather than the bone marrow cells because the engraftment of XY donor derived cells of bone marrow origin has been found to be very poor (<10%) in endometrial glands and stroma [71]. Zhao et al. [72] reported that intrauterine transplantation of autologus bone marrow derived mesenchymal stem cells in women with Asherman’s syndrome resulted in conception. Similarly, Alawadhi et al. [73] reported that transplantation of bone marrow derived stem cells can regenerate mouse model of Asherman’s syndrome and improve fertility rates. Available literature suggests that VSELs are mobilized whenever any organ function is compromised [74-78] and thus endogenous VSELs are expected to be present in the uterus affected by Asherman’s syndrome (similar to their presence in atrophied endometrium in the present study, Gp A). Transplanted bone marrow stem cells act as a source of growth factors/cytokines thus facilitating endogenous VSELs to regenerate the damaged endometrium. In addition, few transplanted stem cells may also get involved in regeneration and thus Y chromosome positive cells in both stromal and epithelial compartment have been reported by Alawadhi et al. [73]. This highlights the fact that a similar stem cell population exists in the endometrium and bone marrow stem cells. Similar to improvement of the thin endometrium by transplanting bone marrow stem cells, surviving endogenous VSELs in chemoablated testis underwent differentiation (spermatogenesis) when a healthy niche was provided by way of transplanting healthy Sertoli or mesenchymal cells [18]. We speculate that if the uterine function of the mice (with Y chromosome positive cells engrafted after transplantation) is compromised by subjecting the mice to bilateral ovariectomy or by any other means (thereby creating regenerative pressure), one may observe clonal expansion, differentiation and increased numbers of Y chromosome bearing cells. Further studies need to be undertaken to delineate endogenous stem cells biology and also Y chromosome bearing cells.

To conclude, we have shown for the first time the presence of two stem cell populations in mouse uterus including pluripotent VSELs and their immediate descendants EnSCs. Data has also been generated to show that these stem cells are modulated by hormones and thus play an important role during endometrial growth and regeneration. VSELs could also be the cancer initiating cells in the endometrium. Further studies are urgently required to study VSELs and EnSCs during endometrial normal biology, cancer and other pathologies.

## **Additional file**

Additional file 1:
**Isolation and flow cytometric analysis of uterine VSELs.**
