# Supplementary material for: Very small embryonic-like stem cells are the elusive mouse endometrial stem cells- a pilot study
Source: J Ovarian Res. 2015 Mar 11;8:9. doi: 10.1186/s13048-015-0138-2 (PMC4369871; doi:10.1186/s13048-015-0138-2)
Supplement: Additional file 1: — Isolation and flow cytometric analysis of uterine VSELs. [file 13048_2015_138_MOESM1_ESM.docx]

**Supplementary section**

***Isolation and flow cytometric analysis of uterine VSELs***

Whole uterus from C57BL/6 (B6) mice (n=15) were used for FACS analysis. Uterine horns were cut into small pieces with a scalpel blade and passed through 18 gauge needle to obtain a single cell suspension. The cells were further incubated for 45 min at 37°C in 25 ml digestion medium (RPMI1640 supplemented with 1000 U/ml Collagenase IV 17104-019 Gibco® Grand Island, NY USA) and Deoxyribonuclease I from bovine pancreas(1mg/ml) (DN25 Sigma, St. Louis, MO, USA). Cells were then filtered through 40 μm filter (BD Biosciences). Cells were washed twice with PBS by spinning down at 3000g for 5 minute. Cells were resuspended in RPMI+2%FBS and counted by haemocytometer. Appropriate counted cells stained with indicated antibodies in supplementary FACS data file. Flow cytometry was performed using the Flow cytometer (LSR II; Becton Dickinson) and FACS Diva software (BD Biosciences, San Diego, CA, USA).

Cell suspension prepared by enzymatic digestion of normal uterine tissues was subjected to flow cytometry to enumerate VSELs. The cells were stained as follows:

1. **SCA-1** Biotin rat anti-mouse Ly-6A/E SCA-1 (1.5µg/5millions cells; 553334; BD Biosciences, USA),Streptavidin-Phycoerythrin-Cy5 (0.6µg/5millions ; 554062; BD)
2. APC-Cy™7 rat anti-mouse **CD45** (0.8µg/5millions; 557659; BD)
3. **Lineage antibody cocktail** as follows,

PE Rat antimouse Ly-6G and Ly-6C(0.4µg/5million; 553128; BD),

PE Rat antimouse CD11b (0.4µg /5millions; 557397; BD),

PE Rat antimouse CD45R/B220 (0.4µg /5millions ; 553089; BD),

PE HamsterantimouseTCR β chain (0.4µg /5millions; 533172; BD)

PE HamsterantimouseTCRγδ (0.4µg /5millions ; 553178; BD)

PE Rat antimouse TER-119/Erythroid cells (0.4µg /5millions; 553673; BD),

The stained cells were run on FACS Aria and the results were analyzed using FACS Diva software (BD). Cells in the size range of 3-5 µm were further gated according to the strategy shown down in Figure. Briefly calibration beads of size 2-15 µm was used according to manufacturer’s instructions (Flow Cytometry Size Calibration Kit microspheres (Invitrogen; Molecular Probes) as reference for selecting cells (P2) ranging from 3–5 µm from total viable population (P1) and to set threshold accordingly. LIN negative & SCA+ cells population (P3) was gated from P2 representing 3–5 µm size cells. Further CD45 negative cells (P4) were gated from LIN negative & SCA+ cells population (P3).

VSELs comprised 3-5 µm cells which were LIN-/CD45-/SCA+( P4 gate)


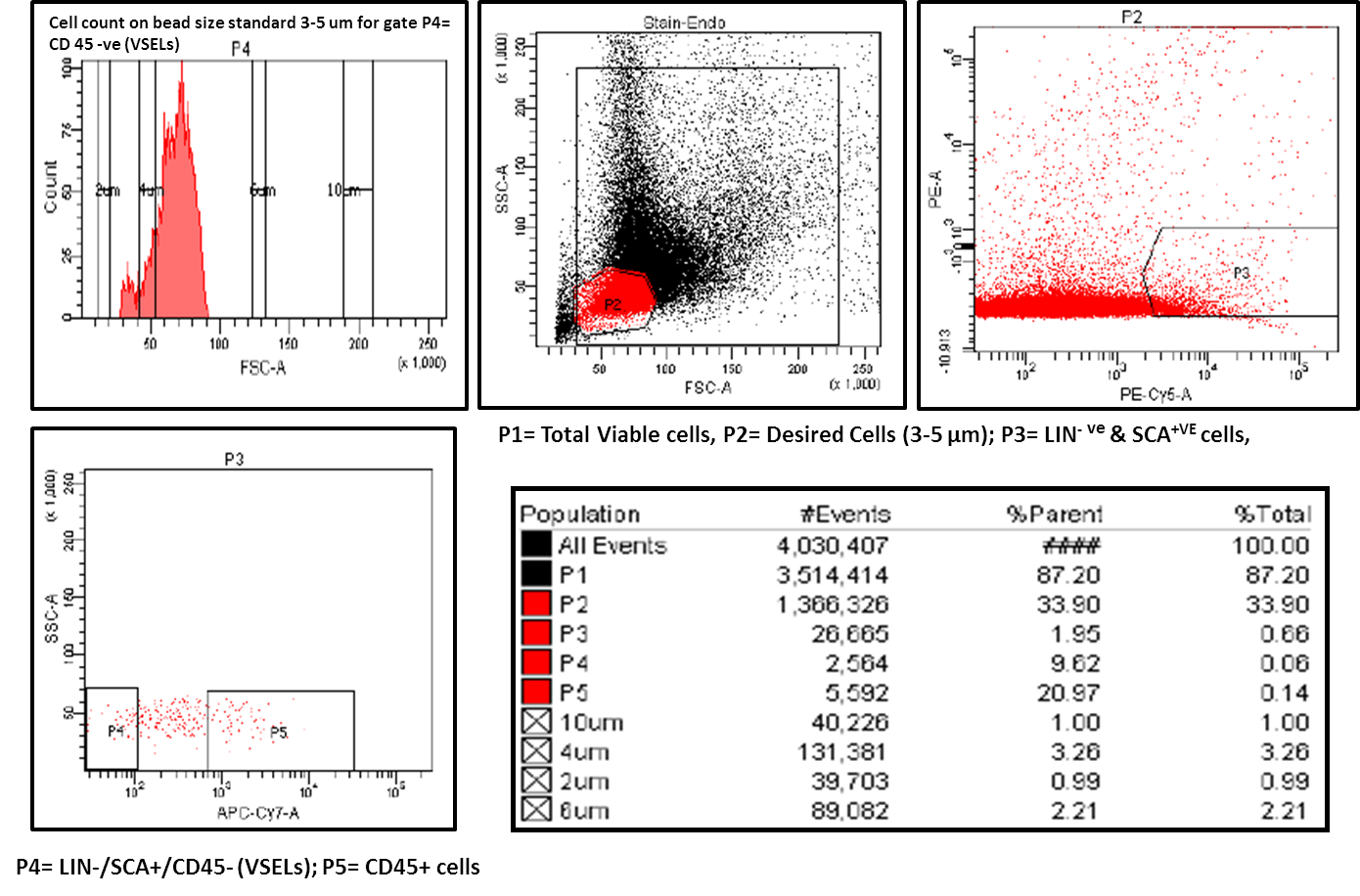


**Results:** Flow cytometry analysis shows the presence of 3–5 µm VSELs which are LIN-/CD45-/SCA-1+ and comprise 0.0685 ± 0.00371 % of uterine cells.

***Western blot analysis for OCT-4 in bilaterally ovariectomized uterus***

Protein was extracted by homogenizing uterine horns in ice cold lysis buffer (50 mM Tris, pH 7.4, 1% NP40, 0.4% sodium deoxycholate, 0.1% SDS, 0.15 M NaCl, 1 mM EDTA, 2 mM PMSF and 1X protease inhibitor cocktail). The cell lysate was agitated on ice for 30 mins followed by centrifugation to collect the supernatant. Protein concentration was estimated by Folin Lowry method using spectrophotometer (Beckman Coulter Inc, IN, USA).Western blotting was carried out based on the standard protocol as summarized below. The extracted protein was incubated in Laemmli buffer for 10 minutes at 95°C. 20-30 μg of protein was loaded onto 10% or 15% SDS-PAGE followed by transfer onto PVDF membrane (Amersham Biosciences, Bucks, UK). The blot was blocked with 5% NFDM in 1X TBST 2 hours at room temperature and then incubated with primary OCT-4 antibody overnight at 4˚C. Membrane was washed with TBST and incubated with biotinylated secondary anti rabbit antibody for 30 minutes, washed and then incubated with Avidin Biotin Complex for 30 minutes (Vectastain Elite ABC kit, Vector Laboratories Inc, USA), washed three times (5 mins each) with TBST buffer and then color development was done using diaminobenzidine (Biogenex, USA).


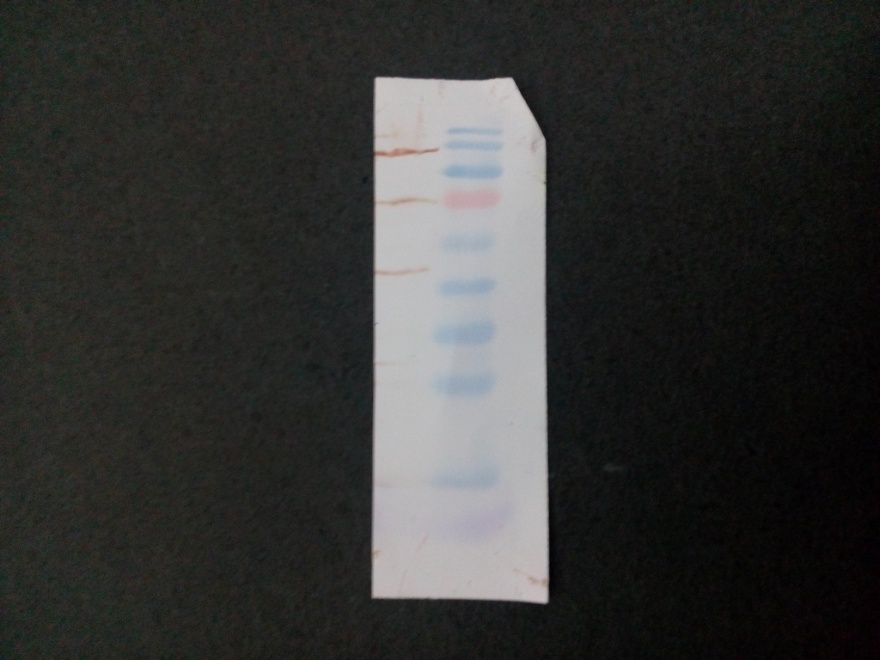


55 kDa

70 kDa

40 kDa

35 kDa

25 kDa

**Results:** Protein bands were observed and nuclear OCT-4A band was obtained around 40 kDa as expected in the protein extracted from uterus belonging to Gp A.
